# What the papers say

**DOI:** 10.1093/jhps/hnag006

**Published:** 2026-02-10

**Authors:** Ali Bajwa

**Affiliations:** Villar Bajwa Practice, Princess Grace Hospital, 30 Devonshire Street, London W1G 6PU, United Kingdom

The *Journal of Hip Preservation Surgery* (*JHPS*) is not the only place where work in the field of hip preservation can be published. Although our aim is to offer the best of the best, we are continually fascinated by work, which finds its way into journals other than our own. There is much to learn from it, and so JHPS has selected six recent and topical subjects for those who seek a summary of what is taking place in our ever-fascinating world of hip preservation. What you see here are the mildly edited abstracts of the original articles, to give them what JHPS hopes is a more readable feel. If you are pushed for time, what follows should take you no more than 10 min to read. So here goes.

## ASSESSING THE QUALITY AND ACCURACY OF CHATGPT-3.5 RESPONSES TO PATIENT QUESTIONS ABOUT HIP ARTHROSCOPY

The authors from Germany [1] note that artificial intelligence-driven large language models such as ChatGPT increasingly influence patient education in surgical fields. This study evaluates the quality and accuracy of ChatGPT-3.5-generated responses to patient questions regarding femoroacetabular impingement syndrome (FAIS) and hip arthroscopy (HAS). In this descriptive observational study, ChatGPT-3.5 generated and answered 20 representative patient questions about FAIS and HAS (*n* = 20 question–answer pairs). No patient-derived questions or data were used. Each response was independently evaluated by two fellowship-trained orthopaedic surgeons across four domains: relevance, accuracy, clarity, and completeness, using a five-point Likert scale. Inter-rater reliability was calculated using the intraclass correlation coefficient (ICC), and descriptive inter-rater agreement percentages were reported. Additional qualitative impressions from the reviewers were recorded to contextualize areas in which responses were rated slightly lower, particularly regarding explanatory depth and postoperative variability.

Mean ratings across all domains ranged from 4.85 (95% CI: 4.74–4.96) to 5.00. Relevance achieved a perfect mean score of 5.00, while accuracy and clarity each obtained 4.98 (95% CI: 4.91–5.00). Completeness demonstrated the lowest scores (4.85). Due to pronounced ceiling effects, ICC values were non-informative; however, descriptive agreement between raters was high, with 100% concordance for relevance and 90% agreement for accuracy and clarity. No factually incorrect or unsafe information was identified. Overall, responses were concise, structured, and clinically appropriate, though occasionally lacking in granularity concerning individual recovery trajectories and procedure-specific nuances. ChatGPT-3.5 demonstrates significant potential as a supplementary patient education tool in hip preservation surgery. While its responses were consistently accurate and easy to understand, their occasional lack of detail, particularly concerning postoperative expectations and variability in outcomes, indicates that the findings apply primarily to synthetic, standardized questions in a controlled setting. Authors concluded that further validation is required before generalizing these results to real-world patient interactions. They stressed that future studies should incorporate authentic patient questions, diverse evaluator groups, and longitudinal assessment across different LLM versions to better define clinical applicability and safety.

## HIP-PRESERVING TREATMENT OF EARLY OSTEONECROSIS OF THE FEMORAL HEAD VIA ARTHROSCOPY: PREOPERATIVE FINITE ELEMENT ANALYSIS PLANNING WITH 3D PRINTING ASSISTANCE

In this study, the authors from China [2] state that osteonecrosis of the femoral head (ONFH) is caused by the disruption of blood flow in the femoral head, leading to irreversible necrosis. Early intervention in ONFH is crucial to prevent femoral head collapse, preserve joint function, and delay or avoid the need for hip replacement. Currently, hip preservation strategies for early ONFH face two significant technical challenges: (a) achieving precision targeting with minimal iatrogenic injury. (b) Precisely identify and target biomechanically optimal 3D location.

This study combined HAS with preoperative finite element mechanical analysis, ‌supplemented by 3D printing technology, aiming to propose a new strategy to guide the precision treatment for early ONFH. Patients treated by this strategy showed a significant decrease in Visual Analogue Scale (VAS) scores from preoperative to 14 days postoperatively, while Harris Hip score (HHS) showed a significant improvement from preoperative to 3-month postoperative follow-up. Of note, imaging results showed that no patient had ONFH progression within 3 years after surgery. Authors felt that this strategy realizes finite element analysis to preoperative planning guidance, practices the biomechanical treatment for early ONFH and worthing further studying.

## HIGH RATES OF RETURN TO AMERICAN FOOTBALL AFTER PRIMARY HIP ARTHROSCOPY FOR FEMOROACETABULAR IMPINGEMENT: A 2-YEAR MINIMUM FOLLOW-UP

Femoroacetabular impingement (FAI) is a common cause of hip pain in young, active athletes. Authors from USA [3] note that American football players place high demands on the hip due to the sport’s requirements for pivoting, cutting, and loading in deep flexion. Although arthroscopic treatment of FAI in professional National Football League (NFL) players has shown favourable outcomes, football-specific outcomes in non-NFL athletes remain limited.

The purpose of their study was to report the return-to-sport (RTS) rates and functional outcomes at a minimum 2-year follow-up for active American football players competing at multiple levels after primary HAS for FAI.

In this case series, level of evidence 4 study, an institutional database was queried to identify all active American football players who underwent primary HAS with labral repair between 2010 and 2023. Demographic characteristics, radiographic parameters, procedure details, complications, and reoperations were collected. Follow-up was completed at a minimum of 2 years to obtain updated patient-reported outcome measures (PROMs) as well as football-specific data, including current participation and RTS status. PROMs were reported as mean ± standard deviation, and categorical variables, including RTS, were reported as frequencies. Pre- versus postoperative PROMs were compared using *t* tests or Mann–Whitney *U* tests to assess outcomes.

In total, 49 hips in 38 male football players (age, 18.1 years; range, 14–26 years) were included. Most athletes played at the high school (74%) or college level (21%). A total of 51% played as linemen. Labral repair was performed in all cases. At a mean 7.6 years, mean modified HHS was 90, Hip Outcome Score-Activities of Daily Living was 95, and Hip Outcome Score-Sport was 89. Overall mean surgery satisfaction was 8.7 (out of 10). Significant postoperative improvements were observed in all PROMs. Twelve players (32%) did not attempt RTS due to other nonmedical factors and were excluded from RTS analysis. A total of 23 (88%) players achieved RTS, and 3 players (12%) did not. Two players (8%) cited other injuries, and 1 player (4%) continued to have hip limitations. Four hips (8%) underwent reoperation at a mean 4.9 years postoperative.

The authors thus concluded that the Amateur American football players undergoing primary HAS for FAI demonstrated excellent patient-reported outcomes (PROs) and a high RTS rate of 88%, when excluding those who did not return for nonmedical reasons, at a mean 7.6-year follow-up. Notable positional differences were observed across multiple levels of play.

## MIDTERM OUTCOMES IN PATIENTS AGED 40 YEARS AND OLDER WITH BORDERLINE DYSPLASIA AFTER HIP ARTHROSCOPY FOR FEMOROACETABULAR IMPINGEMENT: A PROPENSITY-MATCHED ANALYSIS

Age has been shown to play a role in patient outcomes after HAS for the treatment of FAIS, but little is known regarding this relationship in the population with borderline dysplasia. In this study from Chicago, USA [4], authors compare patient outcomes and reoperation rates in patients aged ≥40 years and <40 years with borderline hip dysplasia (BHD) undergoing HAS for FAIS at 2- and 5-year follow-up.

In this level of evidence 3 cohort study, PROs were acquired preoperatively and at 2 and 5 years postoperatively for patients undergoing surgery between January 2012 and June 2019. PROs included the Hip Outcome Score-Activities of Daily Living, Hip Outcome Score-Sports Subscale (HOS-SS), modified HHS, and visual analogue scale for pain and satisfaction. Clinically significant outcomes included the minimal clinically important difference (MCID) and Patient Acceptable Symptom State (PASS). Patients diagnosed with BHD (lateral central-edge angle, 18°–25°) were stratified into cohorts ≥40 and <40 years old. Older patients were propensity matched 1:1 to younger patients controlling for sex, body mass index (BMI), and acetabular cartilage grade. PROs, clinically significant outcomes, and survivorship were compared between groups.

Authors reported that 93 patients aged ≥40 years with BHD (mean age, 48.4 years; 68.8% female; mean BMI, 26.6) were successfully matched to 93 patients aged <40 years with BHD (mean age, 26; 76.3% female; mean BMI, 25.9). Older patients had lower rates of weekly physical activity as compared with younger patients. Older patients with BHD had significantly lower HOS-SS scores than younger patients preoperatively and at 5 years postoperatively. However, there were no differences in improvement of any PRO from presurgery to 5-year follow-up between groups. Older patients had similar achievements of the MCID but achieved the PASS at a significantly lower rate for the HOS-SS (61.1% vs. 79.7%). Older patients also underwent conversion to total hip arthroplasty (THA) at a significantly higher rate (12.8% vs. 1.4%) when compared with younger patients, with a mean conversion time of 4.58 years.

In conclusion, this study notes that patients with BHD aged ≥40 years treated with contemporary HAS for FAIS achieved similar function, pain, and satisfaction at 5-year follow-up but worse sports function and sports-related PASS achievement when compared with patients with BHD aged <40 years. Furthermore, while patients ≥40 years old had a higher rate of THA conversion, they demonstrated similar overall reoperation rates as compared with younger patients at 5 years.

## BIOMECHANICAL COMPARISON BETWEEN INTACT LABRUM, LABRAL TEAR, LABRAL RESECTION, LABRAL REPAIR, AND SEGMENTAL LABRAL RECONSTRUCTION USING FRESH-FROZEN

In this study, DeFroda et al. [5] state that while labral repair is the preferred treatment for labral tears, reconstruction using allografts is an emerging option when repair is not viable. The biomechanical performance of different reconstructive graft options is not fully understood.

Their hypothesis was that segmental labral reconstructions using fresh meniscal allografts (MALs) or fresh-frozen anterior tibialis tendon allografts (TALs) would not be significantly different from native intact labrum with respect to contact areas, contact pressures, peak forces, and suction seal preservation.

In this controlled laboratory study, eight human cadaveric hips were tested using a biomechanical robotic system in intact, tear, repair, resection, and segmental reconstruction with TAL or MAL states. Specimens were examined in neutral, 20° of extension, and 60° of flexion. In each labral state, contact pressure, contact area, and peak force were recorded using pressure sensors. Suction seal distraction tests were performed in each labral state. Data were normalized to the intact labrum state. Repeated-measures analysis of variance was used to identify differences in biomechanical parameters. Significance was set *a priori* at a *P* value <.05.

Segmental labral reconstruction with fresh MAL produced significantly lower peak force than the intact labrum state at 60° of flexion (74.3% ± 13.6% vs. 100%). Segmental reconstruction with fresh-frozen TAL induced a significantly higher peak force at 60° of flexion compared with segmental reconstruction with fresh MAL (133% vs. 74.3%). Intact labrum (7/10; 70%), labral tear (9/10; 90%), labral repair (6/10; 60%), labral resection (1/10; 10%), segmental labral reconstruction with fresh-frozen TAL (4/5; 80%), and segmental labral reconstruction with fresh MAL (4/5; 80%) experienced reestablishment of suction seal after distraction.

The authors concluded that segmental labral reconstruction with fresh MAL resulted in a lower peak force at 60° of flexion and similar rates of suction seal preservation compared with intact labrum. Segmental labral reconstruction with fresh MAL resulted in a lower peak force at 60° of flexion compared with segmental reconstruction with fresh-frozen TAL. Biomechanically, segmental labral reconstruction with fresh MAL may be superior to fresh-frozen TAL in the cadaveric model.

## OBESE PATIENTS TREATED BY HIP ARTHROSCOPY FOR FEMOROACETABULAR IMPINGEMENT SYNDROME-10-YEAR FUNCTIONAL OUTCOMES AND CONVERSION RATES TO ARTHROPLASTY COMPARED WITH NORMAL-WEIGHT PATIENTS

Obesity is a recognized adverse prognostic factor across various surgical interventions. In this study, Quesada-Jimenez et al. [6] evaluate long-term outcomes in patients with obesity who underwent HAS for FAI and labral tears, compared with a control group with normal weight.

This was a prospectively matched cohort study. Data were analysed for patients who underwent primary HAS for FAI and labral tears between October 2008 and October 2013, with a BMI of ≥30 kg/m^2^. Included patients had completed pre- and postoperative PROs and VAS questionnaires at a minimum 10-year follow-up or a documented endpoint within the study time. Rates of revision surgery and survivorship were compared. A subanalysis was performed based on BMI subgroups, and a secondary subanalysis was conducted based on sex. Patients were propensity-matched to a control group of normal-weight patients (BMI, 20–24.99 kg/m^2^) in a 1 to 1 ratio by sex, age at surgery, acetabular outerbridge grade, and capsular treatment.

Authors reported that a total of 266 patients were included in the study, with a mean follow-up time of 125.19 ± 43.07 months. The two groups demonstrated similar magnitudes of improvement at 10-year follow-up for the Non-Arthritic Hip Score (NAHS), Hip Outcome Score-Sport-Specific Subscale (HOS-SSS), and visual analogue scale for pain (VAS-Pain), achieving comparable postoperative scores. The two groups achieved the MCID and PASS for the modified Harris Hip Score (mHHS), NAHS, and HOS-SSS at similar rates. Obese patients had a higher frequency of conversion to THA (odds ratio, 2.19 [95% CI: 1.17–4.13]). Obese patients started with significantly lower baseline preoperative scores for all PROs. Patients with morbid obesity (BMI ≥ 40 kg/m^2^) reached the MCID and PASS for the mHHS at significantly lower rates. No differences in terms of PROs, complications, and secondary surgeries were found in the sex-based subanalysis.

The authors thus concluded that the HAS for the treatment of FAI and labral tears in patients with obesity yielded significant and sustainable long-term improvements, which were equivalent to those of a benchmark matched control group of normal-weight patients. However, patients with obesity had >two-fold odds of conversion to THA. Patients with morbid obesity achieved clinical thresholds at lower rates and should therefore be approached with caution.
